# Psychometric evaluation of the Danish language version of the field practice experiences questionnaire for students in teacher education (FPE-DK) using item analysis according to the Rasch model

**DOI:** 10.1371/journal.pone.0258459

**Published:** 2021-10-18

**Authors:** Tine Nielsen

**Affiliations:** Department of Applied Research in Education and Social Sciences, UCL University College [UCL Erhvervsakademi og Professionshoejskole], Odense, Denmark; Aalborg University, DENMARK

## Abstract

The aim of the study was to conduct a first validation of three field practice experience scales intended to measure students’ opportunities to learn through observation of other teachers, own practice and feedback on own practice of 12 key teaching activities while in field practice placement as part of teacher education programs. The scales were translated and adapted from the elementary teaching candidate survey from the Development of Ambitious Instruction project. Items were adapted to refer to the teaching subject students were training in, and the response scale was modified. A four-step translation-back-translation strategy was used, and subsequently the Danish and a Norwegian and Icelandic translations were mutually adjusted for meaning to facilitate later cross-Nordic studies. Participants were 345 Danish students in the teacher education program from one university college, who had been in at least one field practice placement. Data were collected using a targeted online survey during one month immediately following field placement. Data was analysed using the Rasch model. Each of the three field experience scales fitted a Rasch model, with no evidence against overall homogeneity of scores for low versus high scoring students, local dependence between items, or DIF in relation to level of field practice, campus, type of teacher education program, gender or age. Reliability of each scale was excellent for most subgroups, while the targeting of the scales to the study sample was not very good, as there were too few teaching activities occurring rarely during field practice (i.e. too few difficult items). For all three scales there were significant differences in mean scores dependent on level of field practice placement. Thus, while the scales should be expanded to get better coverage of students’ opportunities to learn in relation to all the core teaching activities present in that are to be trained in the field practice placement, the very good psychometric properties of the three scales, shows promise for future research.

## 1. Introduction

The practice of core skills and activities is an essential part of higher professional education whether this be education toward the health professions (e.g. doctor or nurse) or the educational professions (e.g. school teacher). Within many such educational programs aimed at a profession, these core skills and activities are acquired through field placement and often several field placements to the professional context, where core skills and professions-specific reasoning of increasing complexity are learned through increasing involvement in each field placement. The learning that takes place goes under various names within the health education programs, for example work-integrated learning, experiential learning, workplace-based learning, service learning, clinical learning and so on. What is common is the extensive body on research on this type of learning within many subareas of medical and nursing education and professions [1–3]. With regard to the (school) teacher profession, the terms employed for this type of learning is also varied. Common terms are for example field work learning, teacher practicum, student teaching, and even the term clinical learning is used by some to signal the differences to the “on-campus” and more theoretical learning in the teacher education programs. Within this study the term “learning in field practice placement” will be used, as this reflects the objectives of the Danish teacher education to have field practice separate from campus, that it is a practise situation and that placements are organized by the institutions.

In many countries, teacher education consists of a teaching/education degree on top of a subject Bachelor degree [4]. However, in Denmark as in the other Nordic countries, teacher education is a so-called integrated program focusing on coherence between the teaching subjects, the pedagogical and didactical subjects, and the field practise placements, within a single teacher education program [4, 5]. The Nordic teacher degrees vary in length from four years (Denmark and Sweden) to five years (Norway, Finland and Iceland), and in some of the countries it is also possible to complete a teaching/education in a variety of ways. For example, in Denmark it is possible to do a 2.5 year long teaching degree on top of previous relevant education within a teaching area, in Sweden it is possible to expand the degree to 4.5 years, and in Iceland it is possible to do a 2-year teaching degree on top of a subject degree of relevance [4–6].

Just as the teaching degrees differ across the Nordic countries, so do the field practice placements. In Denmark and Sweden field practise placement amounts to 30 ECTS (European Credit Transfer System), in Finland 20 ECTS, and in Norway it amounts to a minimum of 115 days [4]. In Iceland there are no official regulations on field practice placements, but at the same time there are no teacher education programs without field practise placement (Source: e-mail correspondence with the Icelandic Ministry of Education, Science and Culture) For example, field practice placement within the teacher program at the University of Iceland amounts to 38 ECTS. In Denmark, the field practice placements are regulated from the Ministry of Education and Research, and it is stated explicitly that field practice placement should be offered within the teaching subjects chosen by the students. There can be as many as six field practice placements, but independent of the number of placements these should demonstrate an education-wise progression corresponding to the nationally defined skills and knowledge objectives within three defined areas of competence for each of three levels of field practice. The competence areas and their skills and knowledge objectives for each level of field practise are described in the S1 File. In the teacher education program included in the present study there are three field practice placements corresponding to the three levels of field practice objectives.

Opportunities to practice and learn the core skills of a teacher, can be construed in different ways and thus placed in relation to different part of teacher education programs. One possibility is to enact core skills and activities within the on-campus parts of teacher education [7, 8], for example in the form of role playing, teaching of fellow students, training various micro activities and so on. Such an enactment approach does provide students with an opportunity to practice activities approximating real teaching activities, but in a controlled and artificial setting where they do not teach pupils but co-students and where all the possible unexpected situations and disturbances occurring in the real school context are absent. This is thus a training and learning-wise safe environment to practise in, but at the same time the ecological validity of the learning setting will vary from non-existent to relatively good, depending on the degree of realism installed in the situation. Another, and ecologically valid, possibility to practise and learning core skills is through field practice placement(s), where students can practice in the real context, but under supervision and guidance, in order to both train core skills and learn to cope in unexpected situations. That the ecological aspect of the training context is important is supported by Ronfeldt [9], who in the US context found that later teacher effectiveness and retention was not related to the proportion of poor, minority, or low-achieving pupils in the schools where training was done. However, as also shown by Ronfeldt and colleagues [10], the ecological validity of the field placement environment is not in itself enough to ensure the best outcome, as the teacher students were instructionally more effective, when practising with cooperating teachers that were instructionally effective. Thus, the role model aspect also comes into play in field practice.

Opportunities to learn is a broad construct. It covers opportunities to learn by engaging in real life teaching activities in field practice placement at one end of the spectrum. At the other end of the spectrum, it includes the various parts of a subject curriculum that students in teacher education programs have the opportunity to learn on campus. Focusing on the training of skills, Cohen and Berlin [11] divide opportunities to learn into opportunities to engage with representations and decompositions of practice and opportunities to approximate or enact teaching practices. In the current study, it is attempted to measure both aspects of opportunities to learn as these take place in the field practice placements: Representations of practice through opportunities to learn through observing other teachers, enactment of teaching practice though own practise, and decompositions of practise through feedback on own practice.

The Development of Ambitious Instruction (DAI) Study (www.daiproject.weebly.com), which is an ongoing research project, has developed extensive questionnaires for elementary school teacher candidates enrolled in teacher preparation, their cooperating teachers (i.e. in field practice) and their university supervisors, some of which were also used by Cohen and Berlin [11]. The elementary teaching candidate survey contains multiple sections to survey the teacher candidates educational background, their experience of the teacher preparation in general, and their experience of specified opportunities to learn particularly directed towards teaching English language or mathematics and both in the program in general and specifically during field practise placements. Two sections of this questionnaire assessed whether and how the students experienced opportunities to learn 11 essential teacher activities related to teaching mathematics or English language while in field practice. As these sections appeared readily modifiable in terms of addressing other any teaching subjects, they were found to be of particular interest in relation to an ongoing Nordic collaboration on coherence in teacher education, of which the author is a part. The elementary teaching candidate survey was only available. Thus, in order to utilize it for advancing the field of research on opportunities to learn in teacher education field practice in the Danish and Nordic context, the questionnaire was in need of translation, adaptation as well as rigorous validation.

In the translation and adaptation of existing survey scales to be used both within and across cultures in various language versions, it is important to both ensure measurement invariance across relevant student subgroups within a culture/country as to not bias subsequent sub groups comparisons within the country-setting, as well as to establish first meaning-wise equivalence and then measurement invariance across the language versions as to not bias subsequent comparisons across countries [12–17].

### 1.1 The current study

The aim of the study was to conduct a first investigation of the validity and psychometric properties of the Danish translation of three field practise experience scales for use with Danish students in teacher education programs to assess opportunities to practice and learn crucial professional activities through observation, own practice, and the receiving of feedback. Thus, the purpose of the current study is purely within cultural, as preparation for cross-cultural studies (c.f. the method section).

To fulfil the purpose and aim of the study, Rasch measurement models [18] as well as stratified comparisons of means scores were used for investigation of construct validity and the relationship between scales scores and levels of field practice, respectively. The class of Rasch models readily allow for analyses of local dependence between items and differential item functioning [19], which both impacts on the measurement properties of a scale [14, 20]. Specifically, the below research questions were answered:

- Are the items within each of the three field practice experience scales conditionally independent given the latent variable measures?
- Are the three scales measurement invariant (i.e. free of DIF) across student subgroups defined by the level of the students latest completed field-practice placement as well as other relevant subgroup divisions of students.
- Are the three scales well-targeted and reliable for the study population?
- How are the students’ opportunities to learn associated with the level of their latest field-practice placement targeted in the study?

## 2. Methods

### 2.1. Participants and data collection

The target population was Danish students, who had completed at least one field practice placement as part of their four-year long teacher education program at one Danish university college. The Danish teacher education program includes three field practice placements with common learning objectives across university colleges. Data were collected using a targeted online survey during the month of December 2020. In order to only target students who were likely to have completed at least one field practice placement in the teacher education program, the survey was distributed to 979 students. The data collection resulted in a sample of 404 students, thus the response rate was 41,2%. The study sample consisted of 345 students, who completed the three scales on field practice experiences (c.f. section 2.2. on the instrument) that the current study is concerned with. The reason for the lower number of students in the current study sample was most likely that these scales were placed late in the survey.

The study sample consisted mostly of students enrolled in the regular Bachelor of Education program (84.9%) at one of the two campi (74.2% versus 25.8%) of the university college (Table 1). The distribution across campi in the study sample matched the distribution in the distribution sample (72.7% campus A). The majority of the sample identified as female (70.7%), which was somewhat higher than in the distribution sample (61.4%), and the mean age of the sample was 26.6 years, which matched the distribution sample mean age of 26.8 years. Within the survey, the students also provided information on their major teaching subject, which usually has to be Danish, mathematics or English for primary school or lower secondary school (c.f. Table 1). Also, within the survey students informed on the level of their latest field practice; with the largest groups having completed level 3 field practice (40%) as their latest, while 33.6% had completed level 2 as their latest, and thus 26.4% had completed level 1 field practice as their latest (Table 1).

**Table 1:** Characteristics of the study sample (N = 345).

	Frequency (%)
Campus	
Campus A	256 (74.2)
Campus B	89 (25.8)
BA education program	
Regular	293 (84.9)
other	52 (15.1)
Major teaching subject^a^	
Danish (grade 1–6)	57 (16.5)
Danish (grade 4–10)	110 (31.9)
Mathematics (grade 1–6)	16 (4.6)
Mathematics (grade 4–10)	116 (33.6)
English (grade 1–6)	8 (2.3)
English (grade 4–10)	36 (10.4)
Latest field practice placement	
Level 3	138 (40.0)
Level 2	116 (33.6)
Level 1	91 (26.4)
Gender	
Female	244 (70.7)
Male	101 (29.3)
Age groups	
24 years and younger	172 (49.9)
25 years and older	173 (50.1)
Mean Age (SD), range	26.6 (6.5), 19–58

^a.^ Two students with other Major teaching subject than these six.

Field practice is placed within the first, third and fourth years of study and at varying times in the academic year in the university college in question. The vast majority (90%) of participating students completed their latest field practice placement in 2020. Of the 345 students in the study sample, only 59 (17.1%) completed their latest field practice under special circumstances due to Covid-19 –all of these had completed level 1 field practice as their latest. The circumstances for these students were such that they had 2–3 weeks of virtual field practice where they were either teaching online or otherwise engaging in the school activities from home, due to lockdown of the primary schools. The circumstances of the level 2 and level 3 field practice in 2020 did not differ from the usual modus in significant ways, except that very few students who contracted Covid-19 worked from home for the period of quarantine.

### 2.2. Instrument

The three scales investigated in the current study are Danish translations and slight adaptations of section C6 of the elementary teaching candidate survey from the Development of Ambitious Instruction (DAI) project (available at www.daiproject.weebly.com). Section C6 in the elementary teaching candidate survey contains 11 items with learning activities or opportunities to learn, which can take place during the students’ field-practice placements. The original items were directed towards teaching mathematics, and had a response scale where students could mark each activity/opportunity as experienced in several ways; i.e.”observed other teachers used his practise with students”,”used this practice with students once or twice”,”used this practice with students three or more times”,”received feedback on my attempts to use this practice with students” and”none of the above”. Each occurrence would be counted as one point on the summed scale score, which thus had a score range of 0 to 33, as logically students could not mark both”used this practice with students once or twice”, and”used this practice with students three or more times”, and”none of the above” did not count. Cohen and Berlin [11] shows the item texts, when directed towards students becoming mathematics or English language teachers, with the original response scale and directed towards the teacher preparation program in general rather than just the field practice parts.

Prior to translation, all items were changed to that they did not refer to teaching mathematics, but instead referred to subject-specific teaching or to have no specific reference apart from the reference to field practice teaching in the overall question. The Danish translation was done as part of a larger collaboration with Icelandic and Norwegian researchers in preparation for a cross-Scandinavian research project (c.f. the acknowledgments section), and thus the translation was done in agreement between the three countries. The translation process from English to Danish was done as a forward-backward translation with the following steps:

- Firstly, two independent forward translations were done by two native Danish speaking subject matter experts.
- Secondly, the two translations were compared and evaluated to form a consensus version by a third Native Danish speaking subject matter and psychometric expert.
- Thirdly, the consensus version was back-translated by a Native (American) English speaker fluent in Danish and compared to the original version. This did not result in any changes to the consensus version, as discrepancies were only to allow for differences in the structure of the teacher education program in Denmark.
- Fourthly, the consensus version was evaluated with regards to clarity in relation to the Danish teacher education program by a student, and a few minor adjustments of single words were made.

In order to facilitate future cross-cultural comparison studies and prevent bias caused by differences in translations, a detailed item-level comparison of the phrasing of the Danish translation, a Norwegian, and an Icelandic translation, was conducted by two researchers from each country, so that each country could compare all language versions [13]. Based on the results, the phrasing of a number of items were adjusted in one, two or all three languages, to maximize content and meaning equivalence, while taking into account the existing differences in the teacher education programs of the three countries [16].

Furthermore, the group of researchers decided to change the response format so that each experience of an activity or opportunity to learn would count towards three scales measuring different modes of experience with these. The three modes were derived from the original response formats and were:”I observed other teachers use this practise with students”,”I used this practice with students” and”I received feedback on my attempts to use this practice with students”. Within each of these three modes, students could then reply “no”, “yes, once”, “yes, several times”. The resulting response format was a matrix format, where students at the overall level were asked about the opportunities they had for the various modes of each activity/opportunity to learn during their latest field practice (see S1 Table and S1 Fig, for the item texts in Danish and English as well as the matrix question design for the three scales).

In addition to the 11 items stemming from the DAI project, a 12^th^ item on “facilitation of a good socio-emotional learning environment” was included on the suggestion of the group of Norwegian researchers to tap into the more relational side of classroom management. The three resulting 12-item field practice experience scales were named Observed scale, Practiced scale and Received feedback scale to signal the type of learning opportunities the students experience through the teaching-related activities in field practice placement. The instrument was named FPE-DK for Field Practice Experience–Danish Language version.

### 2.3. Item analyses by graphical log-linear Rasch models and Rasch models

Item analyses were conducted in order to investigate in detail the psychometric properties and validity of each of the three field practice experience scales. Conventional IRT and Rasch models (RMs) assume that items in a scale measure a unidimensional construct, are monotonic, locally independent and invariant without differential item functioning (DIF) of any kind [21]. These four requirements for measurement scales, define criterion-related construct validity according to Rosenbaum [22]. In addition, the RM assumes that item responses are homogeneous [21]. The five requirements are explained and exemplified below:

1. *Unidimensionality*: The items of a scale assess one single underlying latent construct. In this study, the three field practice experience scales each assess one, and only one, experience construct; Observed, Practiced, and Received feedback.
2. *Monotonicity*: The expected item scores on a scale increase with increasing values of the latent variable. In this study for example that the probability having observed another teacher engaged in any of the field practice activities described in the items should increase with increasing scores on the Observed scale.
3. *Local independence of items (no local dependence; No LD)*: The response to a single item should be conditionally independent from the response given to another item of the scale given the latent variable. In this study for example that responses to any one of the items in the Observation scale should depend only on the level of Observation in the field practice and not also on responses to the other items in the scale.
4. *Absence of differential item functioning (no DIF)*: Items and exogenous (i.e. background variables) should be conditionally independent given the latent variable. In this study for example that responses to any one item on the Observed scale should only depend on the level of Observation in the field practice, and not also on subgroup membership (i.e. gender or the level of the field practice).
5. *Homogeneity*: The rank order of the item parameters (i.e. the item difficulties) should be the same across all persons regardless of their level on the latent variable. Thus, for example in this study the items which require the relatively lowest and highest levels of experience with opportunities to learn through observation of other teachers should be the same for all students no matter their level of experience.

It is well-known that both DIF and local dependence of items can affect the psychometric properties of a scale negatively (e.g. biased estimates of the person parameters and inflated alpha estimates; [14, 20]). Since both local dependence and DIF are common problems in non-ability scales and because we prefer to avoid eliminating items for these reasons if the item content appears to be sound these issues were emphasized in the item analyses by attempting to fit item responses to the graphical log-linear Rasch models (GLLRMs) defined by Kreiner and Christensen [23–25]

Graphical log-linear Rasch models replace the requirements of no DIF and local independence with requirements of uniform DIF and uniform local dependence. DIF is uniform if the effect of exogenous variables is the same for all levels of the person parameter [26]. In the same way, local dependence is uniform if the association between locally dependent items is constant across all levels of the person parameter.

Compared to other IRT models, the RM is characterized by the sufficiency of the raw score for the person parameter. Thus, estimation of parameters and inference concerning the fit of items to the RM may be conditional. Conditional inference eliminates person parameters using the *conditional* distributions of items that do not depend on the person parameters. For this reason, conditional inference does not need assumption concerning the distribution or sampling of persons [21, 27].

In GLLRMs, the raw score over items from GLLRMs is also sufficient for the person parameters and conditional inference applies in the same way as for conventional RMs. This property is unique for scales fitting the RM and GLLRMs compared to other item response theory models [21, 25], and it is considered an attractive property in relation to scales such as the ones in this study, where the sum score is used for assessment.

#### 2.3.1. Statistics

Item parameters in GLLRMs and estimates of interaction parameters describing local dependence and DIF are calculated using the same kind of conditional maximum likelihood estimates proposed by Andersen (1970) for the conventional RMs, and assessment of fit to GLLRMs use the same fit statistics that applies for the RM.

Two overall tests of fit were conducted using Andersen conditional likelihood ratio test (CLR) [28]: One was a test of global homogeneity by comparison of item parameters in low and high scoring groups. The other was a global tests of no DIF across the entire set of items in a scale, i.e. a test of overall invariance.).

Individual item fit to the model was tested by comparing the observed item-rest-score correlations with the expected item-restscore correlations under the model (Kreiner & Christensen, 2004)—item-rest-score correlations are correlations between and item and the score with the item excluded.

Local independence of items and DIF at the items level was tested using Kelderman’s likelihood-ratio test [29].

Person fit was assessed using Martin-Löf’s [30] exact test to identify improbable response patterns. The Martin-Löf test uses the conditional distribution of the response pattern given the total score as the test statistic in the same way as in Fisher’s exact test in 2x2 tables. Conditioning with the sufficient score avoids using estimates of person parameters for the test of person fit.

To test the assumption that the three field practice experience scales could be a single unidimensional scale measuring opportunities to learn, pairwise tests of the hypothesis of unidimensionality were conducted for the three scales. The test used compares the observed and expected correlations between subscale scores supposed to depend on different latent variables. The test rejects the hypothesis of unidimensionality if the observed correlation is weaker than the expected correlation under a unidimensional model [31].

Since items, background variables used for testing DIF, and scores are binary and ordinal categorical variables, Goodman & Kruskal’s Gamma (γ) [32] was used to assess the partial correlations among items, items and background variables, and scores. The best way to interpret γ is as a non-parametric correlation coefficient. The definition is similar to Kendall’s Tau except that ties are handled in a better way by γ.

Both the reliability of measurement by the three scales and the targeting of the study populations by the three scales were assessed. Reliability was calculated of the variance of the true score over the variance of the score, and as this depends both on the population and on any DIF among items, reliability was calculated in subgroups defined by DIF-variables and background variables with a significant effect on the score. Targeting of a population by a measure is good if information is high (or SEM is low) for the majority of the persons [33].Targeting of the resulting Rasch scales (i.e. the logit scales of estimated person parameters under the model) was assessed numerically with two indices [33]: the test information target index (the mean test information divided by the maximum test information) and the root mean squared error target index (the minimum standard error of measurement divided by the mean standard error of measurement). Both indices should have a value close to one. The targeting of each scale was illustrated graphically by plotting item maps showing the distribution of item threshold locations against weighted maximum likelihood estimations of the person parameter locations, the person parameters for the population assuming a normal distribution, and the information function. Lastly, the target of the observed scores and the standard error of measurement of the observed scores were calculated.

All the statistical tests used tested whether the item response data complied with the expectations of the model in question, and the results were all evaluated in the same manner with significant p-values providing evidence *against* the model. P-values were evaluated as a continuous measure of evidence distinguishing between weak (p < 0.05), moderate (p < 0.01), and strong (p < 0.001) evidence against the model, as recommended by Cox and colleagues [34], rather than applying a deterministic critical limit of 5%. In addition, the Benjamini-Hochberg procedure [35] was applied to adjust the false discovery rate (FDR) taking multiple testing into account, when appropriate.

#### 2.3.2. Strategy of analysis

Prior to analyses, items were dichotomized to reflect whether the students had experienced opportunities to learn the various core teaching activities in the field practice placements or not. Thus, the two yes response options were collapsed into one category, and thus all items were coded 0 for no opportunity to learn and 1 for opportunity to learn.

Since the RM can be considered the most parsimonious GLLRM (i.e. a GLLRM without DIF or locally dependent items), the following strategy of analyses was used for each of the three field practice experience scales. First, fit of the item responses to each scale was tested rigorously against the RM requirements, as an iterative process aimed at discovering any kind of evidence against the model. If fit to the RM failed because of evidence of DIF and local dependence, the analysis proceeded in a stepwise manner by adding or deleting interaction terms until fit was established. In addition to the tests of local dependence of DIF, overall tests of fit and item fit statistics were calculated during each step and fit was only accepted for a model where no evidence of misfit were disclosed. The below steps were included:

- Overall test of homogeneity of item parameters for low and high scoring groups.
- Overall and item-specific tests of no differential item functioning (no DIF) in relation to the level of Field practice placement (level 3, level 2, level 1), Campus (A, B), Bachelor of Education program (regular, other), Gender (female, male), and Age group (24 years and younger, 25 years and older). For exogenous variables where a subgroup was substantially larger than the other(s), the largest group was used as the reference group (e.g. Field practice placement level 3 and Female gender identification).
- Tests of local independence for all item pairs.
- Fit of the individual items to the model.
- Fit of persons to the model.

After resolving the final model for each of the subscales, targeting and reliability of each subscale was assessed. Finally, it was tested whether the three field practice experience scales made up a single unidimensional opportunities to learn scale.

### 2.4. The relationship between scale scores and level of field practice

The relationship between the students’ scores on the three field practice experience scales (Observed, Practiced, Received feedback) and the level of the students latest completed field practice placement was assessed in two different ways:

1. It was tested whether level of field practice placement was significantly associated with each of the scale scores within the framework of graphical Rasch models [36]. Graphical Rasch models extend the Rasch model (i.e. the measurement model) with a graphical model that includes associations between the score and the exogenous variables, not included in the Rasch model itself. This is useful for assessment of criterion validity or simple associations, while retaining the Rasch model. The magnitude of associations in the graphical Rasch models are informed with partial Goodman and Kruskal’s gamma correlations [32] taking into account any other associations in the model.
2. It was tested whether students’ mean scores of each of the three field practice experience scales differed significantly dependent on level of field practice placement. For this a stepwise analysis of pairwise collapsibility of the mean scores across the three ordinal levels of field practice was used. This procedure is essentially a stepwise and pairwise t-test procedure, where categories with the largest p-values are collapsed into a single category, if the p-value is larger than the set critical p-value (i.e. it is accepted that the mean scores in two categories are statistically equal). Thus for three categories, it might be that two categories are collapsed in the first step, and then this new category is compared to the remaining third category these may or may not be collapsed. This is done, while controlling the false detection rate for multiple testing at the 5% critical level using the Benjamini-Hochberg procedure [35], also used in the item analyses by Rasch models.

### 2.5. Software

All item analysis was conducted using the Digram software package [37, 38]. Item maps and were created using R. Analyses of association and the pairwise collapsibility analyses were done using Digram.

## 3. Results

### 3.1. Item analyses by Rasch models

Using the dichotomized item response data, all three field practice experience scales each fitted a RM. There was no confirmed evidence against overall homogeneity or global DIF relative to the level of latest field practice, the teacher education program students were enrolled in, the campus students were attending, gender or age (Table 2). There was no evidence against the fit of individual items in any of the scales (S2 Table). Furthermore, the tests of no local dependence between items within each of the scales, revealed no evidence of local dependence (S3 Table). The tests of no DIF at the item level revealed no evidence of DIF (S4 Table). Finally, there was no evidence against person fit beyond what could be expected (S5 Table).

**Table 2:** Global tests of homogeneity and differential item functioning for the three field work experience scales.

Tests of fit	Observed scale			Practised scale			Received feedback scale		
	CLR	df	p	CLR	df	p	CLR	df	p
Global homogeneity^d^	16.0	11	.143	9.7	11	.555	14.0	11	.235
Global DIF									
Field practice level	39.6	22	.012^+^	37.7	22	.020^+^	17.5	22	.734
Teacher program	17.3	11	.100	105.6	11	^++^	16.5	11	.125
Campus	13.3	11	.277	9.6	11	.566	10.1	11	.523
Gender	10.7	11	.468	27.0	11	.005^+^	10.7	11	.470
Age	16.1	11	.138	19.7	11	.049	12.6	11	.321

CLR = Conditional Likelihood Ratio test

^d^ The test of homogeneity is a test of the hypothesis that item parameters are the same for persons with low or high scores.

^+^ The Benjamini-Hochberg adjusted critical level for false discovery rate at the 5% level was p = .0083 and at the 1% level was p = .0017.

^++^ no convergence, but no evidence of DIF when analysing at the item level (c.f. S3 Table).

The ranges of the item difficulties for the three field practice experience scales were very similar and relative narrow, though with a slightly wider range for the Observed scale; -1.69 to 0.98 versus -1.18 to 0.97 and -1.26 to 1.00 for the Practiced and Received feedback scales, respectively (Table 3). There was no agreement across the three scales with regard to the relative ordering of items by item difficulty. However, item 10 (*Manage time and student behaviour*) was the easiest item (i.e. required the lowest level on the scale to be endorsed) in both the Observed scale and the Received feedback scale, while the easiest item in the Practiced scale was item 3 (*Differentiate instruction*). The hardest item in both the Observed scale and the Practiced scale was item 5 (*Connect subject specific content to students’ personal/cultural background*), while the hardest item in the Received feedback scale was item 7 (*Facilitate students’ use of technology*).

**Table 3:** Item parameters^a^ for items in the three field work experience scales.

items	Observed scale	Practiced scale	Received feedback scale
1. Design high cognitive demand tasks for students	0.57	0.12	-0.41
2. Teach strategies for learning subject specific content	0.79	0.83	0.54
3. Differentiate instruction	0.15	-1.18	-0.94
4. Connect subject specific content to students’ prior knowledge	0.01	-0.52	-0.47
5. Connect subject specific content to students’ personal/cultural	0.98	1.00	0.96
6. Use representations/models/examples to develop students’ understanding	0.39	0.00	0.21
7. Facilitate students’ use of technology	0.52	0.97	1.00
8. Identify and respond to student thinking	-0.38	-0.84	-0.18
9. Facilitate classroom discussion	-1.01	-0.52	-0.26
10. Manage time and student behaviour	-1.69	-0.92	-1.26
11. Facilitate socio-emotional learning environment *	-0.48	0.51	0.28
12. Provide students subject specific feedback	0.12	0.54	0.52

^a.^ Item parameters (i.e. difficulties, thresholds or locations) represent the point on the latent scale where there is a 50% probability of responding yes to an item, and where item information is maximised.

**Fig 1:**
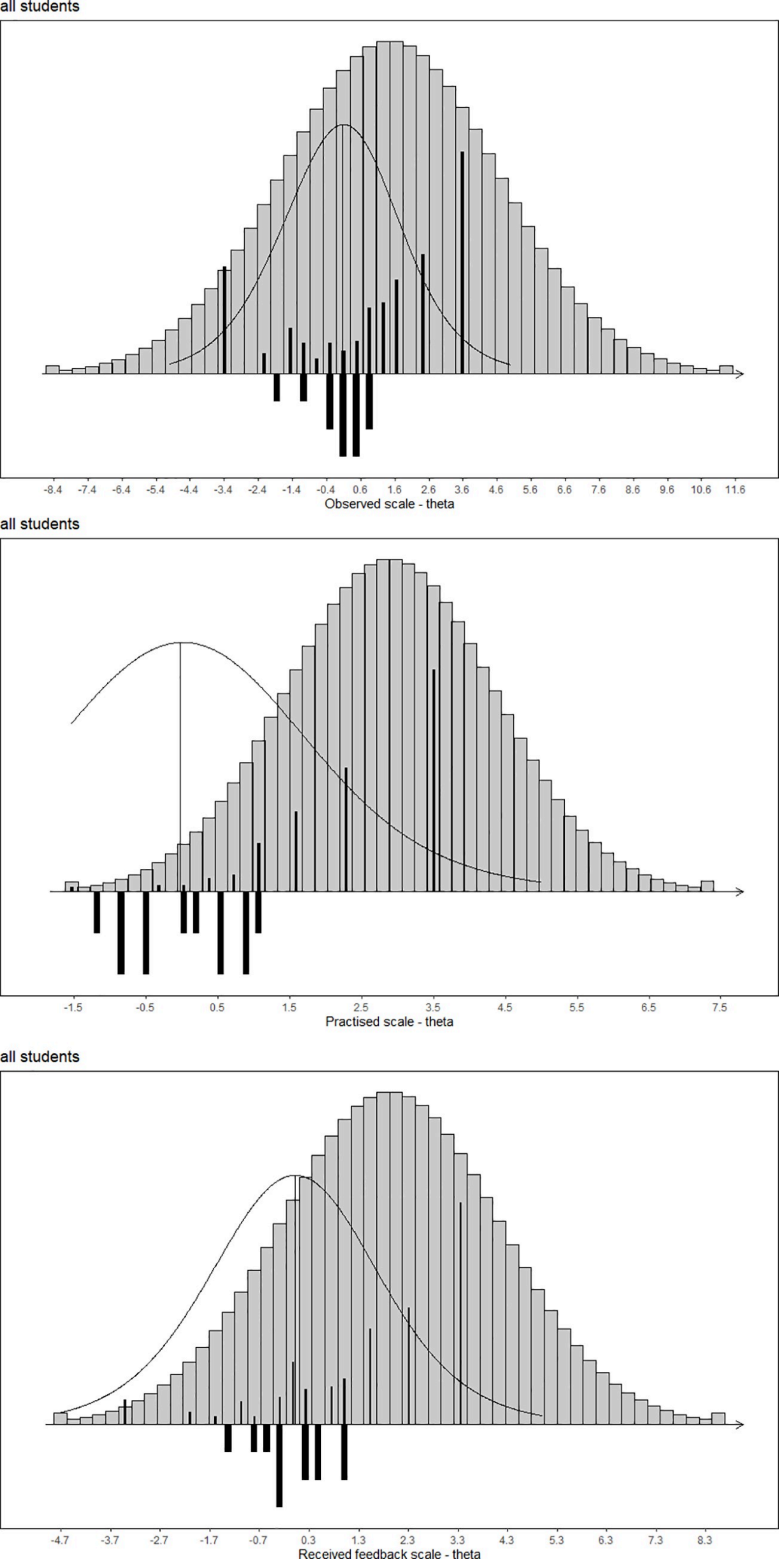
Item maps with distributions of person parameter locations and information curve above item threshold locations for the three field work experience scales; Observed scale (top), Practised scale (middle), Received feedback scale (bottom).

The targeting of the three field practice experience scales was not very good, as less than 60% of the maximum information obtained on average for the subgroups of students defined by level of latest field practice placement and gender (Table 4). The worst targeting all together was the Practiced scale and the received feedback scale for the group of male students in level 3 field practice with less than 20% of the maximum information obtained on average). The best targeting all together was the Received feedback scale for four of the student subgroups with more than 50% of the maximum information obtained on average. The item maps in Fig 1 illustrate how, even though the person estimates for the majority of the student sample are aligned with the item parameters, quite a few students have scores outside the range of the scale where most information is obtained. For the Observed scale, some students score lower or higher than the range with information, while for the Practiced scale and the Received feedback scale no students are below, only above, the range with information. The item maps demonstrate clearly that more “difficult” items are required in order to improve targeting of the three the scales. In the context of students experiencing specific opportunities to learn, this might be construed as opportunities to learn which are not so readily or frequently available as the teaching activities making up the items in the scales. By comparing the teaching activities making up the item content with the nationally set skills objectives for the three field practice level in the teacher education program in Denmark, it is obvious that there are four skills objectives that are not covered at all in by the items in the three scales (S1 File). These are three skills objectives (one for each level of field practice) belonging to the competence domain “relational work” and reflecting skills needed for school-home collaboration, as well as one skill objective belonging to the competence domain “didactics” reflecting the skills needed for developing your own practice and the practice of others based on empirical evidence.

**Table 4:** Targeting and reliability of the three field work experience scales.

	Theta								Sum score			
Student subgroups ^a^ (n)	target	mean	TI mean	TI max	TI Target index	RMSE mean	RMSE min	RMSE target index	target	mean	mean SEM	r^b^
Observed scale												
FP level 1, female (62)	0.10	2.17	1.273	2.675	0.476	0.949	0.611	0.645	6.19	9.42	1.03	0.85
FP level 2, female (82)	0.10	1.31	1.327	2.675	0.496	1.009	0.611	0.606	6.19	7.89	1.05	0.92
FP level 3, female (100)	0.10	0.03	1.247	2.675	0.466	1.106	0.611	0.553	6.19	5.99	1.00	0.94
FP level 1, male (29)	0.10	2.06	1.153	2.675	0.431	1.201	0.611	0.509	6.19	8.93	0.94	0.92
FP level 2, male (34)	0.10	2.91	0.975	2.675	0.365	1.339	0.611	0.437	6.19	9.68	0.83	0.91
FP level 3, male (38)	0.10	1.34	0.916	2.675	0.342	1.822	0.611	0.336	6.19	7.42	0.78	0.96
Practised scale												
FP level 1, female	0.02	2.33	1.226	2.636	0.465	0.871	0.616	0.707	6.03	10.02	1.03	0.77
FP level 2, female	0.02	2.84	0.944	2.636	0.358	0.939	0.616	0.656	6.03	10.65	0.89	0.69
FP level 3, female	0.02	2.75	0.952	2.636	0.361	0.876	0.616	0.703	6.03	10.76	0.92	0.59
FP level 1, male	0.02	2.44	1.154	2.636	0.438	0.849	0.616	0.725	6.03	10.28	1.01	0.66
FP level 2, male	0.02	2.84	0.799	2.636	0.303	0.828	0.616	0.744	6.03	11.09	0.97	0.23
FP level 3, male	0.02	6.69	0.306	2.636	0.116	3.781	0.616	0.163	6.03	11.42	0.34	0.66
Received feedback scale												
FP level 1, female	0.02	1.33	1.517	2.702	0.562	0.851	0.608	0.715	6.04	8.18	1.15	0.89
FP level 2, female	0.02	1.87	1.272	2.702	0.471	1.037	0.608	0.586	6.04	8.71	1.01	0.91
FP level 3, female	0.02	1.80	1.438	2.702	0.532	0.856	0.608	0.711	6.04	9.11	1.12	0.85
FP level 1, male	0.02	1.53	1.590	2.702	0.585	0.792	0.608	0.768	6.04	8.79	1.20	0.93
FP level 2, male	0.02	1.98	1.399	2.702	0.518	0.831	0.608	0.732	6.04	9.53	1.11	0.80
FP level 3, male	0.02	5.16	0.536	2.702	0.199	2.845	0.608	0.214	6.04	10.68	0.51	0.83

TI = test information, RMSE = The root mean squared error of the estimated theta score. SEM = The standard error of measurement of the observed score. r = reliability. FP = Field Practice.

**Fig 2:**
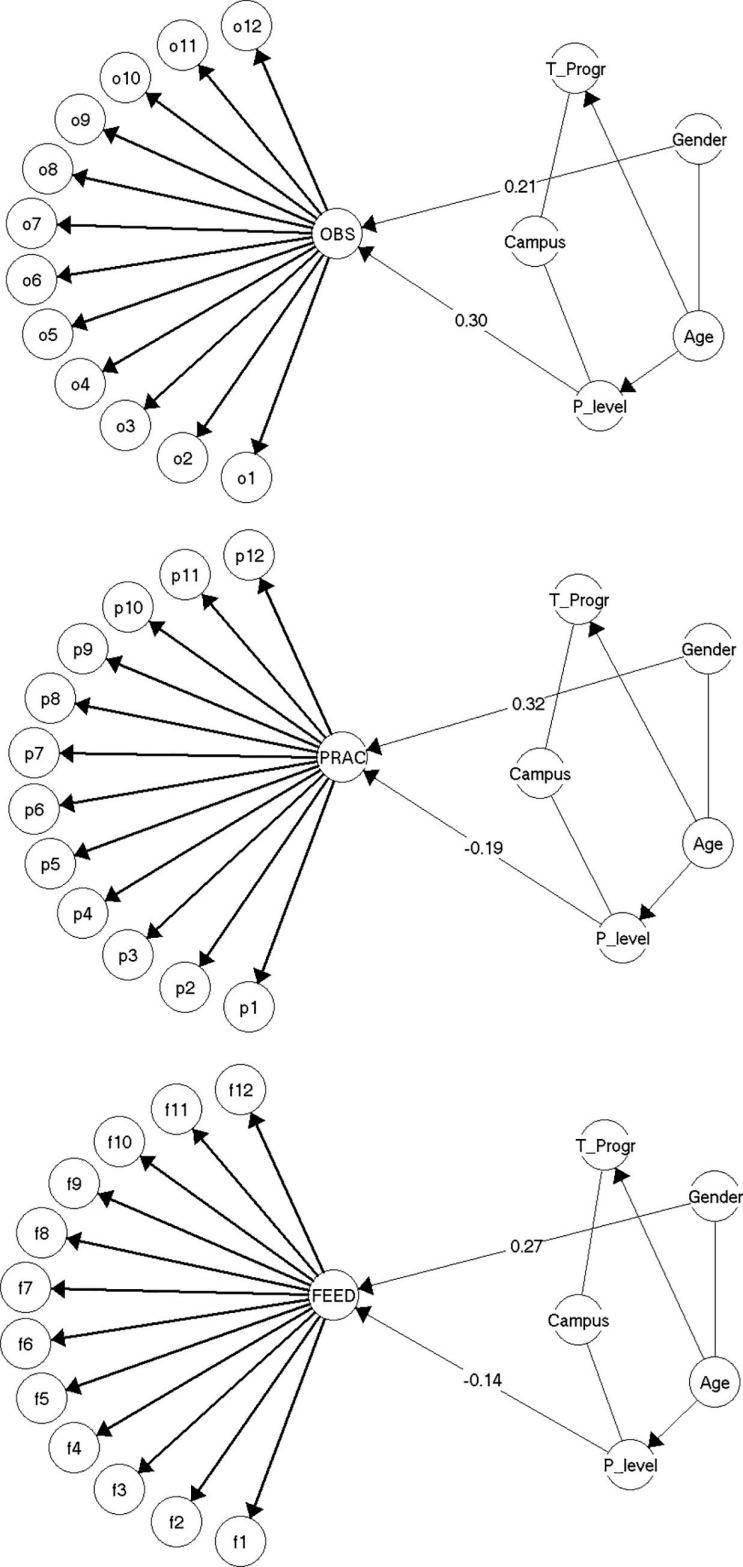
The graphical Rasch models for the three field practice experience scales: Observed scale (top), Practised scale (middle), Received feedback scale (bottom).

^a.^ Targeting and reliability result are provided for subgroups defined by background variables with a significant effect on the respective scale score (c.f. Fig 2).

^b.^ weighted mean reliabilities and person separation for each scale: Observed scale reliability = 0.92, Person separation = 0.80. Practiced scale reliability = 0.65, Person separation = 0.58. Received feedback scale reliability = 0.87, Person separation = 0.75.

The reliability of both the Observed and the Received feedback scale were very good to excellent for all subgroups of student (ranging from 0.83 to 0.96). The reliability of the Practiced subscale was less than acceptable for all student subgroups, except for female students in their level 1 field practice (Table 4). For the group of male students in their level 2 field practice reliability was virtually non-existent.

Person parameters are weighted maximum likelihood estimates and illustrate the distribution of these for the study sample (black bars above the line) and for the population under the assumption of normality (grey bars above the line), as well as the information curve, relative to the distribution of the item difficulties (black bars below the line).

Table 5 shows the results of the pairwise tests of unidimensionality conducted to test whether the three scales make up a single unidimensional opportunities to learn scale. The three subscales are significantly correlated implying that the same is true for the underlying latent traits, but correlations are significantly weaker than expected under unidimensionality. Thus, the notion of a unidimensional opportunities to learn scale is rejected.

**Table 5:** Test of unidimensionality of the field practice experience scales.

Scales	Obs γ	Exp γ	SE exp γ	asymptotic p	exact p^a^
Observed & Practiced	.274	.726	.024	< .0001	< .0001
Observed & Received feedback	.302	.748	.021	< .0001	< .0001
Practised & Received feedback	.713	.757	.025	.087	<. 01

γ-correlations are Goodman and Kruskal’s rank correlations for ordinal data. Obs γ = observed correlations between scales. Exp γ = expected correlations under the model.

^a.^ Parametric bootstrapping with 1000 samples.

The S6 Table in the supporting information provides the summed scale scores as well as the person parameter estimates resulting from the RMs–the so-called Rasch scores–in order to facilitate conversion from one to the other for researchers wanting to do that. Standard errors of measurement (SEM) for both the summed scale scores and the person parameter estimates are also shown. It is evident that measurement error is large for both the summed score and the person parameter estimates in all three scales, though it varies somewhat along the scales.

### 3.2. The relationship between scale scores and levels of field practise placement

#### 3.2.1. The graphical Rasch models

In the graphical Rasch models, which also include association between the respective scale score and the, to the Rasch model, exogenous background variables, it was found that each of the three field practice experience scale scores were correlated to both the level of the latest field practice placement and the gender of the students, but with different directions and strengths of the partial correlations for each scale (Fig 2). For the Observed scale there was a strong negative relationship between the level of field practice placement and the Observed scale score, so that the *lower* the level of the students’ latest field practice placement, the higher they would score on the Observed scale (γ = 0.30). In addition, male students score *higher* on the observed scale compared to female students (γ = 0.21). For the Practised scale and the Received feedback scale the relationship between the level of field practice placement and the respective scale scores was instead positive and not as strong as for the Observed scale correlation between gender. Thus, the *higher* level of field practice placement, the higher score on the Practised and the Received feedback scales (γ_Practised_ = -0.19, γ_Received feedback_ = -0.14). With regard to the associations with gender male students score *higher* on the Practised and the Received feedback scale compared to female students (γ_Practiced_ = 0.32, γ_Received feedback_ = 0.27).

γ-correlations are partial Goodman and Kruskal’s rank correlation for ordinal data. Level of field practice placement (P_level) is coded in reverse (c.f. the method section) and thus correlations should be interpreted with opposite signs.

#### 3.2.2. Differences in mean scale scores

As both the summed scale scores and the Rasch scores had substantial measurement error, and the summed scale score facilitated direct interpretation of the number of opportunities to learn in each of the three modes of experience (Observed, Practised and Received feedback), the summed scale score was used for comparison across levels of field practice. Table 6 shows the mean scores on each of the three field practice experience scales are shown for all the students and stratified by field practice level (S2 Fig shows the score distributions). Results of the stepwise analysis of pairwise collapsibility of the mean scores across levels of field practice are shown in Table 7. With regard to the Observed scale, equality of the mean scores for students in field practice level 1 and level 2 (see Table 6) was accepted and thus the two groups were collapsed to a single group with a common mean score on the Observed scale (8.86). Equality of this common mean score for students in field practice 1 and 2 and lower the mean score for students in level 3 field practice was strongly rejected (p < 0.001). With the Received feedback scores, these were also found to be equal for students in level 1 and 2 field practice, and that the common mean (8.69) was not equal to the higher mean score for students in level 3 field practice, though with weaker evidence against equality (p < 0.05). Lastly, with the Practiced scale score, the mean scores for students in level 2 and level 3 field practice were found to be equal, and the common mean score for these students (10.87) to differed from the slightly lower mean score of students in level 1 field practice (p < 0.01).

**Table 6:** Mean scale scores, standard deviations and 95% confidence intervals for all students and stratified by level of field practice placement (N = 345).

	Observed scale			Practiced scale			Received feedback scale		
	Mean	SD	95% CI	Mean	SD	95% CI	Mean	SD	95% CI
All students	7.82	4.26	[7.37; 8.28]	10.66	1.82	[10.46; 10.86]	9.03	3.34	[8.68; 9.39]
Field practice level 1	9.26	3.29	[8.58; 9.94]	10.09	2.18	[9.64; 10.55]	8.37	3.47	[7.65; 9.09]
Field practice level 2	8.41	3.93	[7.69; 9.13]	10.78	1.59	[10.48; 11.07]	8.94	3.43	[8.32; 9.58]
Field practice level 3	6.38	4.67	[5.59; 7.17]	10.94	1.66	[10.47; 10.86]	9.54	3.11	[9.02; 10.07]

CI = Confidence Interval.

**Table 7:** Common mean scale scores for students in collapsed groups of field practice levels (N = 345).

	Observed scale		Practiced scale		Received feedback scale	
Field practice	Mean	SE	Mean	SE	Mean	SE
level 1	8.86	0.25	10.09	0.23	8.69	0.24
level 2			10.87	0.10		
level 3	6.38	0.39			9.54	0.26

The Benjamini-Hochberg procedure was used to control the false discovery rate due to multiple comparisons.

## 4. Discussion and implications

The results of the current study show promise with regards to being able to measure students’ opportunities to learn crucial teacher activities through observation, through own practice and through receiving feedback while in field practice placements in the teacher education program. As this is the first study of these adapted scales, it is not possible to discuss the results against previous research. Thus, the discussion will primarily be directed more toward the psychometric properties of the scales and the implications these have for use of the scales and further development/research.

### 4.1. The psychometric properties of the scales

The results showed that the three field practice experience scales each fitted a Rasch model when item were dichotomized to reflect whether students had experienced the various opportunities to learn. Fit to the Rasch model is desirable for the context in which the scales are intended, as this allows practitioners (and researcher) to use the summed scale score to assess the degree to which students experience opportunities to learn through the three modes of observation, own practice and feedback, as this is sufficient for the estimated Rasch scores resulting from the model–and naturally, the latter can also be used at the practitioners or researchers discretion, by using the conversion tables (S6 Table). While no evidence of departures from the Rasch model requirements could be detected by the many tests and statistics used, there are still parts of the results in need of discussion.

The fact that no evidence against absence of differential item functioning (i.e. measurement invariance), both when tested for the entire set of items with the global tests and for individual items with item-specific tests, does not necessarily mean that there is no DIF. Thus, it might be that one or more of the scales could suffer from DIF relative to other subgroups of students than the ones included in this study (i.e. gender, age, campus, teacher education program and level of field practice). It might also be that evidence against absence of DIF could have been detected with a larger sample, as the sample size for this first study is modest. What the study does show, is that for modest sample sizes, which might be common in institution-wise studies of students’ field practice experiences, there was no evidence against measurement invariance, and thus any DIF would have to quite substantial to be evident in larger samples. The first recommendation for future studies is thus that these should include larger samples and institutions-wise a wider selection of students, in order to confirm or disprove the present findings as well as elaborate on these.

No evidence against conditional independence of the items was discovered for any of the three scales. As conditional independence of items is a requirement for all IRT models, and one that is rarely met–if investigated, this is a positive finding. The reliability of the three scales for the current study sample varied both across the scales and within each scale for the subgroups of students defined by gender and the level of field practice. Reliability was the highest for the Observed scale and the Received feedback scale, and for all but one subgroup on the Observed scale and two subgroups on the Received feedback scale high enough for individual assessment (0.90 or higher) [39, 40], and above 0.80 for the remaining subgroups. Reliability was the lowest for the Practised scale with values between 0.59 and 0.77 for five subgroups, and an extremely low value of 0.23 for the subgroup of male students in level 2 field practice. These low reliability values are most probably due to lack of variation in the Observed scale, as most students reported having had the various opportunities to learn. The reliability results do not call for particular recommendations with regard to future studies, but rather attention to whether this persists with other study samples.

Also related to measurement precision is the issue of targeting of the scale items to the study population, as this was found to be less than satisfactory. The main issue, with all three scales, was a need for items that were, in psychometric terms, harder to endorse, which in this context would entail teacher activities and opportunities to learn occurring less frequently during the field practice placements. Recently it has been shown that misalignment in learning objectives and what is covered by assessments in higher education affect student motivation [41]. However, not only the assessments can be misaligned with learning objectives, so can the opportunities to learn in order to achieve the objectives, and it reasonable to assume that such misalignment would also affect student motivation. Thus, it is crucial that instruments intended to measure students’ opportunities to learn cover the associated learning objectives in their intended context of use, if the instruments should serve practical and research purposes beyond merely measuring opportunities to learn: e.g. studies of student motivation, engagement and self-efficacy as these relate to opportunities to learning, study progress and outcomes. In the current study it is reasonable to assume that the teaching activities reflecting the areas of the national skills objectives not covered by the items in the scales would occur less frequently than the teaching activities in the scale items, as the school-home collaboration can be a sensitive situation where students teachers are not invited as observers or participants, and as the professional development based on empirical evidence can be hard to achieve within the relatively short field practice placements in the Danish teacher education. Therefore, it is recommended that the Danish version of the FPE instrument is extended with activities reflecting the missing skills objectives so that a more encompassing measure of Danish teacher students’ opportunities to learn while in field practice placement. The same type of extension might readily be done in the other language versions of the FPE, while preserving the current 12 core activities in all language versions for cross-cultural comparison studies.

All three scales fitted a Rasch model and sufficiency was obtained for both the sum score and the person parameter estimates. This means that both can be used for the assessment of student experience of the three types of opportunities to learn in filed practise, as all necessary information is contained in both. Some might argue that assessment should be made using the person parameter estimates (i.e. the Rasch scores on the logit scale), as these are considered to be at interval scale-level and thus more accurate in terms of having equal distance between values along the entire scale. However, with using the logit scores all meaning of the scales are lost, as we do not know what a difference in one logit means in regards to opportunities to learn. As sufficiency of the sum score is a property only of scales fitting the Rasch model, there is the advantage of using this instead and retaining the meaning of the scores. This is found to be particularly valuable in the current case, as the three scales are in fact “absolute counting scales”, with each score value giving the number of the particular mode of opportunities to learn that a student has experienced in field practice placement.

### 4.2. Relationship between scale scores and level of field practice

The relationship between level of field practice and the three scale scores was investigated both as correlations within the framework of graphical Rasch models [36] and through comparisons of mean scores for these subgroups. In the graphical Rasch models resulting from the item analyses, it was found that when taking into account all of the five grouping variables, only gender and level of field practice were correlated to the score, and this was the case for all three scales. there was no a-priory expectation of the relationship between level of field practice and the three scale scores, as no previous research on this issue could be identified. It is thus a new finding that level of field practice is negatively and strongly correlated to the Observed scale score, while only moderately and positively correlated to the Practised and the Received feedback scale scores. When comparing the mean scale scores for students in the three levels of field practice, the magnitudes vary and not all three groups differ significantly.

The strong negative correlation between level of field practice and the Observed scale score shows that the students report that they have less and less opportunities to learn through observation of other teachers the higher the level of field practice, and thus also, the more advanced the skills objective are. When comparing the mean scores for students in the three levels of field practise only two differ significantly; the joint group of students in level 1 and level 2 field practice differ from the students in level 3 field practice by 2,5 scale points. This can appear as a desired development as the students progress towards their professional teacher identity. However, it might also be viewed as somewhat problematic from a learning point of view, as the ability to learn from observation is enhanced by the level of knowledge, the level of reflection, and the professional maturity of the students (i.e. vicarious learning beyond imitation and conditioning). Thus, the students should in general be the best equipped for vicarious learning in the highest level of field practice. Whether this finding is a local finding or would also be found in other teacher education programs internationally, should be a subject for future research.

With regard to the moderate positive correlations between level of field practice and the Practised scales scores and the Received feedback scale scores, this was to some degree expected as personal practise and receiving feedback are at the core of the concept of field practice placement in teacher education in Denmark. There is hardly any difference in the mean Practised scale scores according to level of field practise, though the ¾ point difference between students in level 1 field practice from the remaining students is significant, and for both groups the mean score is higher than ten thus confirming that the core purpose of the field practice is fulfilled for the majority of students. Turning to the Received feedback mean scale scores, these differ significantly with approximately 1 ¼ scale point between the group of students in level 1 field practice and the joint groups of students in level 2 and level 3 field practice. What stands out is the fact that it is the students in the higher levels of field practice who report receiving feedback on the most teaching activities. This might simply be due to the fact that not all students having practised an activity will have received feedback, and thus the feedback score could be expected to be lower, or it could be a development across the levels of field practise. As feedback is crucial for development towards being a fully capable teacher, and maximizing such opportunities to learn early appears sensible, this finding should be investigated further in future studies, and in particular in a longitudinal setting, as we cannot from these data determine whether this is a common pattern or if it is a development across the levels of field practise.

## 5. Conclusion

In conclusion, the three field practice experience scales each fitted Rasch models and thus the raw summed score can be used for assessment, as can the person parameter estimates resulting from each model. The three scales, are however, in need of additional items, as targeting was not very good. Particularly, items reflecting teaching activities and opportunities to learn which occur less frequently in students’ field practice placements should be added to the scales (i.e. items, which, in psychometric terms, are more difficult to endorse). In the Danish teacher education context such items might be created taking inspiration in the national skills objectives for the field practices, and should then include teacher activities focusing on self-development as a teacher, the development of others as teachers, and the school-home collaboration. As the scales should be investigated further with larger and, in terms of university colleges, a more diverse study population, new items might successfully be developed for such studies. However, as the instrument is also intended for cross-cultural comparative studies, it may very well be that the instrument should consist of a common cross-cultural part as well as country-specific parts, which cover opportunities to learn that are considered core teacher activities reflected in skills objectives in the specific country. The scales are suited for uncovering relevant differences in the level of opportunities to learn that students’ experience in field practice, and further studies should be undertaken to expand this knowledge.

## Supporting information

S1 Fig The matrix question design for the three scales. (TIF)

S2 Fig Score distributions of the three field practice experience scales. (TIF)

S1 Table The Danish item texts of the three field practice experience scales and their English counterparts. (DOCX)

S2 Table Item fit statistics for the three field practice experience scales. (DOCX)

S3 Table Conditional likelihood ratio tests of local independence for the three field practice experience scales. (DOCX)

S4 Table Conditional likelihood ratio tests of no DIF for the three field practice experience scales. (DOCX)

S5 Table Assessment of person fit of the three field practice experience scales. (DOCX)

S6 Table Weighted maximum likelihood estimates of person parameters for the three field work experience scales. (DOCX)

S1 File Description of competence objectives for field practice in teacher education. (DOCX)
