# Supplementary figures and images for: Psychometric evaluation of the Danish language version of the field practice experiences questionnaire for students in teacher education (FPE-DK) using item analysis according to the Rasch model
Source: PLoS One. 2021 Oct 18;16(10):e0258459. doi: 10.1371/journal.pone.0258459 (PMC8523040; doi:10.1371/journal.pone.0258459)

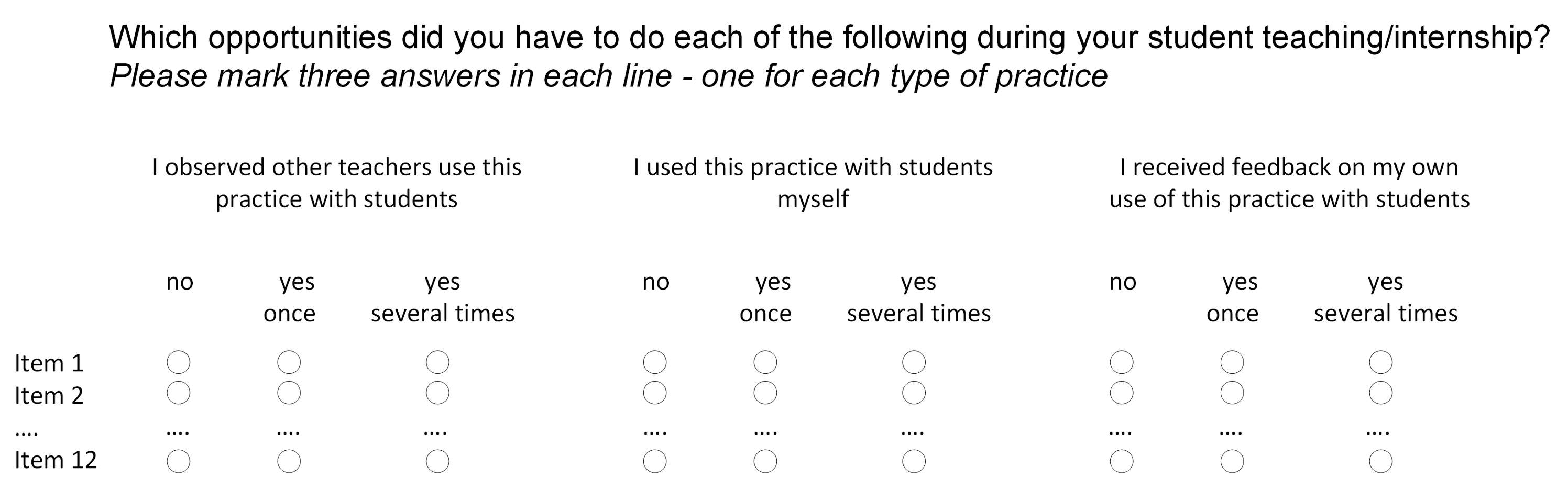

Supplement: S1 Fig — (TIF) [file pone.0258459.s001.tif]

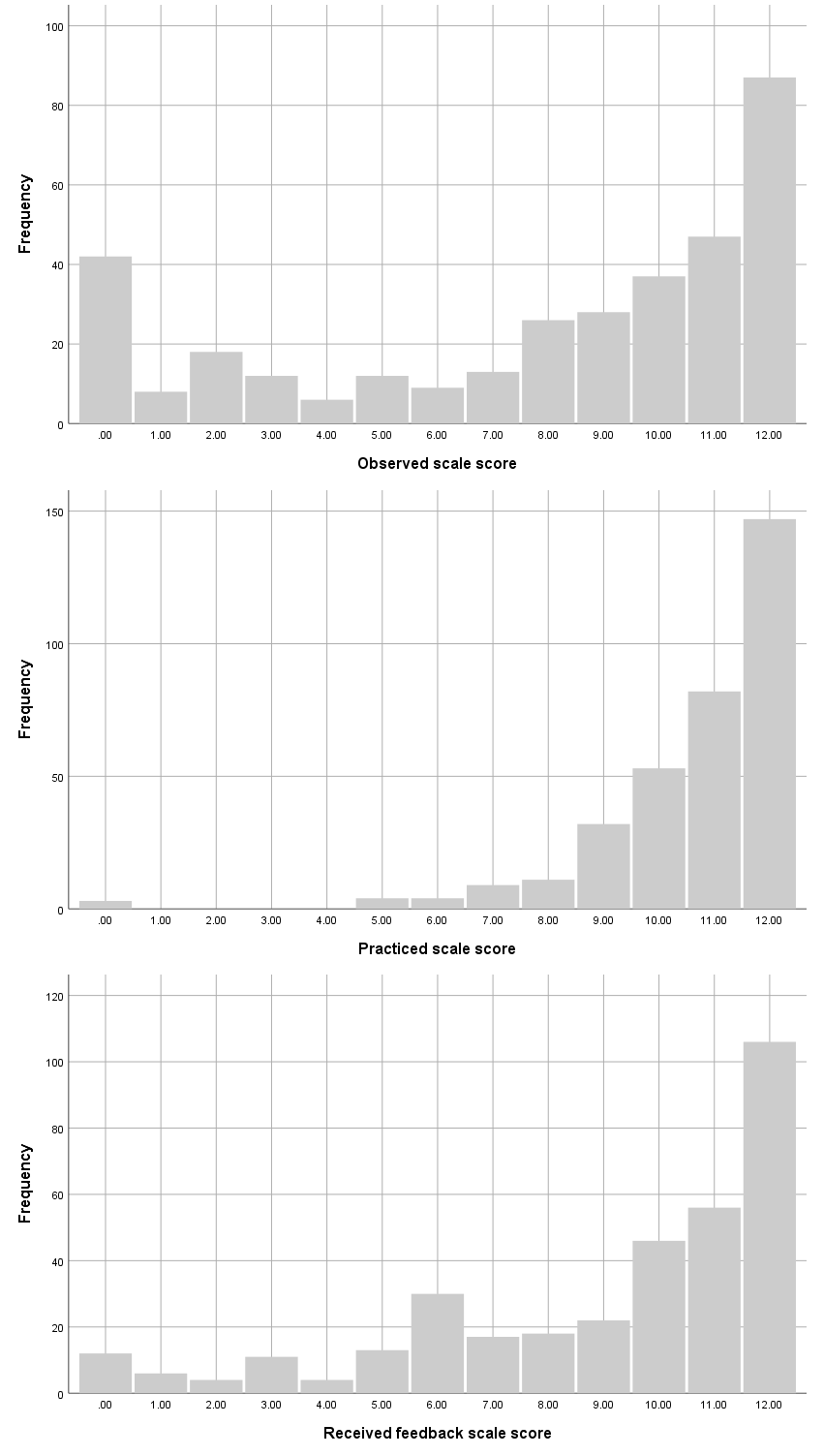

Supplement: S2 Fig — (TIF) [file pone.0258459.s002.tif]
