# Supplementary material for: Psychometric evaluation of the Danish language version of the field practice experiences questionnaire for students in teacher education (FPE-DK) using item analysis according to the Rasch model
Source: PLoS One. 2021 Oct 18;16(10):e0258459. doi: 10.1371/journal.pone.0258459 (PMC8523040; doi:10.1371/journal.pone.0258459)
Supplement: S1 Table — (DOCX) [file pone.0258459.s003.docx]

**S1 Table. The Danish item texts of the three field practice experience scales and their English counterparts^a^.**

| Danish item texts | English item texts |
| --- | --- |
| 1. Planlægge kognitivt krævende opgaver til eleverne | 1. Design high cognitive demand tasks for students |
| 2. Undervise eleverne i strategier til at lære fagspecifikt indhold | 2. Teach strategies for learning subject specific content |
| 3. Differentiere undervisning | 3. Differentiate instruction |
| 4. Forbinde fagspecifikt indhold til elevernes forhåndsviden | 4. Connect subject specific content to students’ prior knowledge |
| 5. Forbinde fagspecifikt indhold til elevernes personlige og kulturelle erfaring | 5. Connect subject specific content to students’ personal/cultural |
| 6. Anvende repræsentationsformer/modeller/eksempler til at udvikle elevernes forståelse | 6. Use representations/models/examples to develop students’ understanding |
| 7. Facilitere elevernes brug af teknologi | 7. Facilitate students’ use of technology |
| 8. Identificere og respondere på elevernes ideer og tænkning | 8. Identify and respond to student thinking |
| 9. Facilitere klassesamtale | 9. Facilitate classroom discussion |
| 10. Udøve klasseledelse (dvs. med fokus på tidsforbrug og elevers adfærd) | 10. Manage time and student behavior |
| 11. Facilitere et godt socioemotionelt læringsmiljø * | 11. Facilitate socio-emotional learning environment * |
| 12. Give eleverne fagspecifik feedback | 12. Provide students subject specific feedback |

^a.^ The English items are not entirely identical to the Danish due to the changes described in the method section.

* This item was added by the Norwegian research group.
