# Supplementary material for: Psychometric evaluation of the Danish language version of the field practice experiences questionnaire for students in teacher education (FPE-DK) using item analysis according to the Rasch model
Source: PLoS One. 2021 Oct 18;16(10):e0258459. doi: 10.1371/journal.pone.0258459 (PMC8523040; doi:10.1371/journal.pone.0258459)
Supplement: S2 Table — (DOCX) [file pone.0258459.s004.docx]

**S2 Table. Item fit statistics for the three field practice experience scales.**

| Observed scale | | | |  | Practiced scale | | |  | Received feedback scale | | |
| --- | --- | --- | --- | --- | --- | --- | --- | --- | --- | --- | --- |
| items | Obs γ | Exp γ | p |  | Obs γ | Exp γ | p |  | Obs γ | Exp γ | p |
| i1 | 0.77 | 0.81 | 0.165 |  | 0.57 | 0.56 | 0.975 |  | 0.66 | 0.75 | 0.043^+^ |
| i2 | 0.78 | 0.81 | 0.394 |  | 0.65 | 0.54 | 0.169 |  | 0.71 | 0.74 | 0.578 |
| i3 | 0.90 | 0.83 | 0.025^+^ |  | 0.58 | 0.64 | 0.649 |  | 0.76 | 0.77 | 0.824 |
| i4 | 0.88 | 0.84 | 0.144 |  | 0.57 | 0.59 | 0.830 |  | 0.76 | 0.76 | 0.967 |
| i5 | 0.84 | 0.80 | 0.271 |  | 0.63 | 0.54 | 0.235 |  | 0.74 | 0.73 | 0.786 |
| i6 | 0.82 | 0.82 | 0.979 |  | 0.55 | 0.57 | 0.869 |  | 0.80 | 0.74 | 0.151 |
| i7 | 0.78 | 0.82 | 0.296 |  | 0.45 | 0.54 | 0.243 |  | 0.67 | 0.73 | 0.181 |
| i8 | 0.89 | 0.85 | 0.280 |  | 0.74 | 0.61 | 0.294 |  | 0.80 | 0.75 | 0.295 |
| i9 | 0.84 | 0.87 | 0.301 |  | 0.38 | 0.59 | 0.052 |  | 0.72 | 0.75 | 0.565 |
| i10 | 0.91 | 0.90 | 0.720 |  | 0.71 | 0.62 | 0.448 |  | 0.88 | 0.79 | 0.081 |
| i11 | 0.82 | 0.85 | 0.211 |  | 0.47 | 0.55 | 0.343 |  | 0.78 | 0.74 | 0.355 |
| i12 | 0.82 | 0.83 | 0.682 |  | 0.56 | 0.55 | 0.888 |  | 0.73 | 0.74 | 0.958 |

Obs = Observed; Exp = Expected under the model.γ = Item-rest-score correlations for the respective Rasch models in Fig 2 and Table 2. γ-correlations are Goodman and Kruskal’s rank correlation for ordinal data.

^+^ Benjamini-Hochberg-adjusted critical level for false discovery rate above the 5% level.
