# Supplementary material for: Psychometric evaluation of the Danish language version of the field practice experiences questionnaire for students in teacher education (FPE-DK) using item analysis according to the Rasch model
Source: PLoS One. 2021 Oct 18;16(10):e0258459. doi: 10.1371/journal.pone.0258459 (PMC8523040; doi:10.1371/journal.pone.0258459)
Supplement: S3 Table — (DOCX) [file pone.0258459.s005.docx]

**S3 Table. Conditional likelihood ratio tests of local independence for the three field practice experience scales.**

|  | Observed scale | | |  | Practiced scale | | |  | Received feedback scale | | |
| --- | --- | --- | --- | --- | --- | --- | --- | --- | --- | --- | --- |
| Item pairs  tested | *CLR* | *df* | *p* |  | *CLR* | *df* | *p* |  | *CLR* | *df* | *p* |
| 1 & 2 | 0.54 | 1 | 0.461 |  | 0.63 | 1 | 0.429 |  | 0.27 | 1 | 0.605 |
| 1 & 3 | 0.01 | 1 | 0.914 |  | 0.08 | 1 | 0.776 |  | 1.42 | 1 | 0.233 |
| 1 & 4 | 1.65 | 1 | 0.199 |  | 0.54 | 1 | 0.464 |  | 5.99 | 1 | 0.014 |
| 1 & 5 | 1.10 | 1 | 0.295 |  | 0.64 | 1 | 0.422 |  | 0.35 | 1 | 0.556 |
| 1 & 6 | 0.99 | 1 | 0.321 |  | 0.02 | 1 | 0.883 |  | 1.03 | 1 | 0.310 |
| 1 & 7 | 3.64 | 1 | 0.056 |  | 0.01 | 1 | 0.936 |  | 5.63 | 1 | 0.018^+^ |
| 1 & 8 | 0.21 | 1 | 0.649 |  | 0.02 | 1 | 0.899 |  | 0.96 | 1 | 0.327 |
| 1 & 9 | 2.77 | 1 | 0.096 |  | 0.22 | 1 | 0.638 |  | 1.89 | 1 | 0.169 |
| 1 & 10 | 0.84 | 1 | 0.360 |  | 0.46 | 1 | 0.498 |  | 1.68 | 1 | 0.195 |
| 1 & 11 | 1.08 | 1 | 0.299 |  | 0.76 | 1 | 0.384 |  | 1.62 | 1 | 0.203 |
| 1 & 12 | 2.34 | 1 | 0.126 |  | 0.16 | 1 | 0.692 |  | 3.08 | 1 | 0.079 |
| 2 & 3 | 0.13 | 1 | 0.717 |  | 0.03 | 1 | 0.858 |  | 1.51 | 1 | 0.219 |
| 2 & 4 | 0.56 | 1 | 0.454 |  | 0.11 | 1 | 0.745 |  | 0.23 | 1 | 0.633 |
| 2 & 5 | 0.24 | 1 | 0.624 |  | 3.77 | 1 | 0.052 |  | 0.05 | 1 | 0.825 |
| 2 & 6 | 0.02 | 1 | 0.897 |  | 2.87 | 1 | 0.091 |  | 3.00 | 1 | 0.083 |
| 2 & 7 | 0.16 | 1 | 0.689 |  | 1.04 | 1 | 0.309 |  | 3.93 | 1 | 0.048^+^ |
| 2 & 8 | 0.06 | 1 | 0.802 |  | 4.93 | 1 | 0.026 |  | 0.21 | 1 | 0.649 |
| 2 & 9 | 4.92 | 1 | 0.027^+^ |  | 0.73 | 1 | 0.394 |  | 0.10 | 1 | 0.746 |
| 2 & 10 | 0.19 | 1 | 0.661 |  | 0.57 | 1 | 0.450 |  | 0.59 | 1 | 0.443 |
| 2 & 11 | 0.49 | 1 | 0.483 |  | 0.01 | 1 | 0.920 |  | 0.03 | 1 | 0.871 |
| 2 & 12 | 9.04 | 1 | 0.003^+^ |  | 0.04 | 1 | 0.839 |  | 1.15 | 1 | 0.283 |
| 3 & 4 | 11.02 | 1 | 0.001^+^ |  | 3.26 | 1 | 0.071 |  | 0.39 | 1 | 0.532 |
| 3 & 5 | 9.47 | 1 | 0.002^+^ |  | 1.49 | 1 | 0.223 |  | 2.20 | 1 | 0.138 |
| 3 & 6 | 6.51 | 1 | 0.011^+^ |  | 1.02 | 1 | 0.313 |  | 0.66 | 1 | 0.415 |
| 3 & 7 | 0.28 | 1 | 0.596 |  | 0.00 | 1 | 0.980 |  | 0.04 | 1 | 0.851 |
| 3 & 8 | 8.58 | 1 | 0.003^+^ |  | 2.43 | 1 | 0.119 |  | 2.13 | 1 | 0.145 |
| 3 & 9 | 0.03 | 1 | 0.852 |  | 0.19 | 1 | 0.660 |  | 3.99 | 1 | 0.046^+^ |
| 3 & 10 | 0.14 | 1 | 0.709 |  | 2.27 | 1 | 0.132 |  | 0.15 | 1 | 0.699 |
| 3 & 11 | 0.11 | 1 | 0.745 |  | 0.62 | 1 | 0.433 |  | 0.05 | 1 | 0.818 |
| 3 & 12 | 6.02 | 1 | 0.014^+^ |  | 2.69 | 1 | 0.101 |  | 0.00 | 1 | 0.958 |
| 4 & 5 | 5.56 | 1 | 0.018^+^ |  | 0.13 | 1 | 0.719 |  | 3.48 | 1 | 0.062 |
| 4 & 6 | 5.29 | 1 | 0.022^+^ |  | 1.56 | 1 | 0.212 |  | 3.89 | 1 | 0.049 |
| 4 & 7 | 0.21 | 1 | 0.645 |  | 0.01 | 1 | 0.925 |  | 0.46 | 1 | 0.498 |
| 4 & 8 | 1.08 | 1 | 0.299 |  | 0.00 | 1 | 0.974 |  | 0.42 | 1 | 0.516 |
| 4 & 9 | 0.80 | 1 | 0.372 |  | 0.09 | 1 | 0.764 |  | 0.63 | 1 | 0.427 |
| 4 & 10 | 1.25 | 1 | 0.264 |  | 0.83 | 1 | 0.361 |  | 4.86 | 1 | 0.028^+^ |
| 4 & 11 | 0.04 | 1 | 0.833 |  | 0.08 | 1 | 0.772 |  | 0.10 | 1 | 0.752 |
| 4 & 12 | 0.03 | 1 | 0.869 |  | 0.09 | 1 | 0.760 |  | 1.25 | 1 | 0.264 |
| 5 & 6 | 0.08 | 1 | 0.778 |  | 0.71 | 1 | 0.390 |  | 0.01 | 1 | 0.931 |
| 5 & 7 | 0.02 | 1 | 0.885 |  | 0.45 | 1 | 0.503 |  | 0.04 | 1 | 0.849 |
| 5 & 8 | 1.53 | 1 | 0.216 |  | 6.15 | 1 | 0.013^+^ |  | 0.05 | 1 | 0.818 |
| 5 & 9 | 1.40 | 1 | 0.237 |  | 5.17 | 1 | 0.023^+^ |  | 1.35 | 1 | 0.245 |
| 5 & 10 | 0.44 | 1 | 0.505 |  | 0.48 | 1 | 0.488 |  | 0.01 | 1 | 0.937 |
| 5 & 11 | 0.01 | 1 | 0.904 |  | 0.01 | 1 | 0.916 |  | 0.42 | 1 | 0.518 |
| 5 & 12 | 0.94 | 1 | 0.333 |  | 2.06 | 1 | 0.151 |  | 0.25 | 1 | 0.614 |
| 6 & 7 | 0.21 | 1 | 0.644 |  | 1.49 | 1 | 0.222 |  | 0.00 | 1 | 0.971 |
| 6 & 8 | 1.93 | 1 | 0.165 |  | 2.21 | 1 | 0.138 |  | 2.20 | 1 | 0.138 |
| 6 & 9 | 2.45 | 1 | 0.117 |  | 3.93 | 1 | 0.048^+^ |  | 0.14 | 1 | 0.706 |
| 6 & 10 | 0.61 | 1 | 0.434 |  | 0.03 | 1 | 0.859 |  | 2.82 | 1 | 0.093 |
| 6 & 11 | 7.88 | 1 | 0.005^+^ |  | 0.01 | 1 | 0.924 |  | 5.47 | 1 | 0.019 |
| 6 & 12 | 0.66 | 1 | 0.415 |  | 0.54 | 1 | 0.462 |  | 0.00 | 1 | 0.978 |
| 7 & 8 | 0.08 | 1 | 0.777 |  | 0.71 | 1 | 0.399 |  | 0.52 | 1 | 0.469 |
| 7 & 9 | 0.64 | 1 | 0.425 |  | 0.36 | 1 | 0.547 |  | 1.71 | 1 | 0.191 |
| 7 & 10 | 0.14 | 1 | 0.707 |  | 2.75 | 1 | 0.097 |  | 0.47 | 1 | 0.492 |
| 7 & 11 | 3.55 | 1 | 0.060 |  | 2.87 | 1 | 0.090 |  | 0.18 | 1 | 0.672 |
| 7 & 12 | 0.15 | 1 | 0.694 |  | 0.19 | 1 | 0.664 |  | 0.03 | 1 | 0.873 |
| 8 & 9 | 1.02 | 1 | 0.313 |  | 0.00 | 1 | 0.974 |  | 0.23 | 1 | 0.635 |
| 8 & 10 | 0.50 | 1 | 0.480 |  | 0.15 | 1 | 0.698 |  | 5.11 | 1 | 0.024^+^ |
| 8 & 11 | 0.27 | 1 | 0.601 |  | 0.26 | 1 | 0.610 |  | 1.41 | 1 | 0.235 |
| 8 & 12 | 1.71 | 1 | 0.191 |  | 0.02 | 1 | 0.890 |  | 1.49 | 1 | 0.222 |
| 9 & 10 | 2.22 | 1 | 0.136 |  | 2.66 | 1 | 0.103 |  | 11.80 | 1 | 0.001^+^ |
| 9 & 11 | 0.03 | 1 | 0.869 |  | 0.06 | 1 | 0.802 |  | 0.94 | 1 | 0.333 |
| 9 & 12 | 2.46 | 1 | 0.117 |  | 4.36 | 1 | 0.037 |  | 0.53 | 1 | 0.467 |
| 10 & 11 | 0.01 | 1 | 0.927 |  | 3.49 | 1 | 0.062 |  | 5.50 | 1 | 0.019^+^ |
| 10 & 12 | 2.92 | 1 | 0.088 |  | 1.69 | 1 | 0.193 |  | 1.83 | 1 | 0.177 |
| 11 & 12 | 0.87 | 1 | 0.351 |  | 0.59 | 1 | 0.442 |  | 0.06 | 1 | 0.809 |

^+^ The Benjamini-Hochberg adjusted critical level for false discovery rate due to multiple testing at the 5% level was p = .0004 for tests of local independence and tests of no DIF, and thus all p-values were considered insignificant.
