# Supplementary material for: Psychometric evaluation of the Danish language version of the field practice experiences questionnaire for students in teacher education (FPE-DK) using item analysis according to the Rasch model
Source: PLoS One. 2021 Oct 18;16(10):e0258459. doi: 10.1371/journal.pone.0258459 (PMC8523040; doi:10.1371/journal.pone.0258459)
Supplement: S4 Table — (DOCX) [file pone.0258459.s006.docx]

**S4 Table. Conditional likelihood ratio tests of no DIF for the three field practice experience scales.**

|  | Observed scale | | |  | Practised scale | | |  | Received feedback scale | | |
| --- | --- | --- | --- | --- | --- | --- | --- | --- | --- | --- | --- |
| Items and exogenous variables tested | *CLR* | *df* | *p* |  | *CLR* | *df* | *p* |  | *CLR* | *df* | *p* |
| 1 & Campus | 0.57 | 1 | 0.450 |  | 1.28 | 1 | 0.259 |  | 0.23 | 1 | 0.633 |
| 2 & Campus | 6.81 | 1 | 0.009^+^ |  | 0.98 | 1 | 0.323 |  | 5.53 | 1 | 0.019^+^ |
| 3 & Campus | 1.85 | 1 | 0.173 |  | 1.18 | 1 | 0.277 |  | 0.11 | 1 | 0.740 |
| 4 & Campus | 0.78 | 1 | 0.377 |  | 0.61 | 1 | 0.436 |  | 0.03 | 1 | 0.852 |
| 5 & Campus | 0.00 | 1 | 0.967 |  | 0.01 | 1 | 0.943 |  | 0.70 | 1 | 0.404 |
| 6 & Campus | 0.32 | 1 | 0.569 |  | 1.07 | 1 | 0.300 |  | 0.49 | 1 | 0.485 |
| 7 & Campus | 1.78 | 1 | 0.182 |  | 0.55 | 1 | 0.460 |  | 0.58 | 1 | 0.448 |
| 8 & Campus | 0.00 | 1 | 0.999 |  | 0.30 | 1 | 0.583 |  | 1.27 | 1 | 0.260 |
| 9 & Campus | 0.06 | 1 | 0.799 |  | 0.96 | 1 | 0.327 |  | 0.18 | 1 | 0.671 |
| 10 & Campus | 0.03 | 1 | 0.865 |  | 2.23 | 1 | 0.136 |  | 0.69 | 1 | 0.405 |
| 11 & Campus | 0.31 | 1 | 0.575 |  | 1.10 | 1 | 0.294 |  | 0.53 | 1 | 0.466 |
| 12 & Campus | 1.61 | 1 | 0.205 |  | 0.08 | 1 | 0.771 |  | 0.74 | 1 | 0.388 |
| 1 & Teacher program | 0.00 | 1 | 0.976 |  | 0.63 | 1 | 0.428 |  | 1.20 | 1 | 0.274 |
| 2 & Teacher program | 3.73 | 1 | 0.053 |  | 0.17 | 1 | 0.682 |  | 1.71 | 1 | 0.191 |
| 3 & Teacher program | 1.04 | 1 | 0.307 |  | 1.94 | 1 | 0.164 |  | 1.50 | 1 | 0.221 |
| 4 & Teacher program | 1.45 | 1 | 0.228 |  | 0.16 | 1 | 0.688 |  | 0.01 | 1 | 0.907 |
| 5 & Teacher program | 0.19 | 1 | 0.663 |  | 0.91 | 1 | 0.339 |  | 0.64 | 1 | 0.423 |
| 6 & Teacher program | 0.17 | 1 | 0.676 |  | 2.70 | 1 | 0.101 |  | 4.30 | 1 | 0.038^+^ |
| 7 & Teacher program | 0.01 | 1 | 0.923 |  | 0.22 | 1 | 0.639 |  | 0.17 | 1 | 0.679 |
| 8 & Teacher program | 0.63 | 1 | 0.427 |  | 0.01 | 1 | 0.933 |  | 1.22 | 1 | 0.269 |
| 9 & Teacher program | 4.89 | 1 | 0.027^+^ |  | 0.12 | 1 | 0.726 |  | 0.02 | 1 | 0.897 |
| 10 & Teacher program | 4.43 | 1 | 0.035 |  | 0.99 | 1 | 0.321 |  | 6.31 | 1 | 0.012 |
| 11 & Teacher program | 0.00 | 1 | 0.977 |  | 0.16 | 1 | 0.686 |  | 0.60 | 1 | 0.438 |
| 12 & Teacher program | 0.00 | 1 | 0.944 |  | 0.38 | 1 | 0.535 |  | 0.21 | 1 | 0.644 |
| 1 & Field work level | 0.17 | 2 | 0.918 |  | 2.01 | 2 | 0.367 |  | 8.29 | 2 | 0.016^+^ |
| 2 & Field work level | 1.81 | 2 | 0.405 |  | 1.57 | 2 | 0.456 |  | 1.59 | 2 | 0.452 |
| 3 & Field work level | 0.27 | 2 | 0.872 |  | 3.63 | 2 | 0.163 |  | 0.81 | 2 | 0.667 |
| 4 & Field work level | 1.89 | 2 | 0.389 |  | 6.14 | 2 | 0.046^+^ |  | 0.06 | 2 | 0.969 |
| 5 & Field work level | 7.87 | 2 | 0.020^+^ |  | 2.51 | 2 | 0.286 |  | 0.88 | 2 | 0.644 |
| 6 & Field work level | 1.87 | 2 | 0.393 |  | 0.02 | 2 | 0.989 |  | 0.06 | 2 | 0.969 |
| 7 & Field work level | 5.07 | 2 | 0.079 |  | 2.65 | 2 | 0.266 |  | 0.52 | 2 | 0.771 |
| 8 & Field work level | 6.63 | 2 | 0.036^+^ |  | 3.20 | 2 | 0.202 |  | 0.13 | 2 | 0.939 |
| 9 & Field work level | 8.44 | 2 | 0.015^+^ |  | 7.18 | 2 | 0.028 |  | 1.41 | 2 | 0.493 |
| 10 & Field work level | 3.72 | 2 | 0.156 |  | 2.30 | 2 | 0.317 |  | 2.10 | 2 | 0.349 |
| 11 & Field work level | 5.10 | 2 | 0.078 |  | 5.58 | 2 | 0.062 |  | 1.42 | 2 | 0.491 |
| 12 & Field work level | 0.01 | 2 | 0.994 |  | 4.12 | 2 | 0.127 |  | 1.68 | 2 | 0.432 |
| 1 & Gender | 0.00 | 1 | 0.981 |  | 7.94 | 1 | 0.005 |  | 0.43 | 1 | 0.511 |
| 2 & Gender | 0.70 | 1 | 0.402 |  | 0.38 | 1 | 0.536 |  | 1.91 | 1 | 0.167 |
| 3 & Gender | 0.63 | 1 | 0.428 |  | 6.80 | 1 | 0.009^+^ |  | 2.19 | 1 | 0.139 |
| 4 & Gender | 1.46 | 1 | 0.226 |  | 0.02 | 1 | 0.810 |  | 0.21 | 1 | 0.646 |
| 5 & Gender | 1.03 | 1 | 0.311 |  | 0.19 | 1 | 0.664 |  | 0.37 | 1 | 0.542 |
| 6 & Gender | 1.19 | 1 | 0.275 |  | 2.77 | 1 | 0.096 |  | 0.46 | 1 | 0.496 |
| 7 & Gender | 0.05 | 1 | 0.829 |  | 0.00 | 1 | 0.963 |  | 0.07 | 1 | 0.793 |
| 8 & Gender | 0.00 | 1 | 0.949 |  | 0.67 | 1 | 0.412 |  | 0.36 | 1 | 0.549 |
| 9 & Gender | 0.85 | 1 | 0.356 |  | 4.57 | 1 | 0.033^+^ |  | 0.26 | 1 | 0.611 |
| 10 & Gender | 0.13 | 1 | 0.717 |  | 0.47 | 1 | 0.493 |  | 0.27 | 1 | 0.602 |
| 11 & Gender | 1.56 | 1 | 0.211 |  | 1.81 | 1 | 0.179 |  | 3.49 | 1 | 0.062 |
| 12 & Gender | 4.07 | 1 | 0.044^+^ |  | 3.35 | 1 | 0.067 |  | 1.66 | 1 | 0.197 |
| 1 & Age | 4.64 | 1 | 0.031^+^ |  | 1.92 | 1 | 0.166 |  | 0.76 | 1 | 0.383 |
| 2 & Age | 0.53 | 1 | 0.469 |  | 0.57 | 1 | 0.450 |  | 1.44 | 1 | 0.230 |
| 3 & Age | 1.16 | 1 | 0.281 |  | 3.87 | 1 | 0.049^+^ |  | 0.86 | 1 | 0.354 |
| 4 & Age | 2.48 | 1 | 0.115 |  | 2.10 | 1 | 0.147 |  | 0.76 | 1 | 0.383 |
| 5 & Age | 3.23 | 1 | 0.073 |  | 0.98 | 1 | 0.322 |  | 0.30 | 1 | 0.581 |
| 6 & Age | 0.01 | 1 | 0.934 |  | 0.81 | 1 | 0.367 |  | 1.41 | 1 | 0.235 |
| 7 & Age | 1.28 | 1 | 0.257 |  | 2.13 | 1 | 0.144 |  | 0.77 | 1 | 0.380 |
| 8 & Age | 1.25 | 1 | 0.264 |  | 2.52 | 1 | 0.113 |  | 1.42 | 1 | 0.233 |
| 9 & Age | 0.52 | 1 | 0.472 |  | 0.21 | 1 | 0.645 |  | 0.00 | 1 | 0.999 |
| 10 & Age | 1.05 | 1 | 0.305 |  | 3.91 | 1 | 0.048 |  | 1.32 | 1 | 0.250 |
| 11 & Age | 0.03 | 1 | 0.854 |  | 1.31 | 1 | 0.252 |  | 0.10 | 1 | 0.752 |
| 12 & Age | 1.42 | 1 | 0.233 |  | 0.89 | 1 | 0.345 |  | 4.68 | 1 | 0.031^+^ |

^+^ The Benjamini-Hochberg adjusted critical level for false discovery rate due to multiple testing at the 5% level was p = .0004 for tests of no DIF and tests of local independence, and thus all p-values were considered insignificant.
