# Supplementary material for: Psychometric evaluation of the Danish language version of the field practice experiences questionnaire for students in teacher education (FPE-DK) using item analysis according to the Rasch model
Source: PLoS One. 2021 Oct 18;16(10):e0258459. doi: 10.1371/journal.pone.0258459 (PMC8523040; doi:10.1371/journal.pone.0258459)
Supplement: S5 Table — (DOCX) [file pone.0258459.s007.docx]

**S5 Table. Assessment of person fit of the three field practice experience scales.**

|  | Observed Scale | | | Practiced Scale | | | Received feedback scale | | |
| --- | --- | --- | --- | --- | --- | --- | --- | --- | --- |
| Score | frequency | misfit frequency | percentage | frequency | misfit frequency | percentage | frequency | misfit frequency | percentage |
| 1 | 8 | 0 | 0.0 | 0 | 0 | 0.0 | 6 | 0 | 0.0 |
| 2 | 18 | 3 | 16.7 | 0 | 0 | 0.0 | 4 | 0 | 0.0 |
| 3 | 12 | 1 | 8.3 | 0 | 0 | 0.0 | 11 | 2 | 18.2 |
| 4 | 6 | 0 | 0.0 | 0 | 0 | 0.0 | 4 | 0 | 0.0 |
| 5 | 12 | 1 | 8.3 | 4 | 1 | 25.0 | 13 | 0 | 0.0 |
| 6 | 9 | 0 | 0.0 | 4 | 0 | 0.0 | 30 | 1 | 3.3 |
| 7 | 13 | 1 | 7.7 | 9 | 0 | 0.0 | 17 | 0 | 0.0 |
| 8 | 26 | 2 | 7.7 | 11 | 0 | 0.0 | 18 | 3 | 16.7 |
| 9 | 28 | 1 | 3.6 | 32 | 1 | 3.1 | 22 | 1 | 4.5 |
| 10 | 37 | 2 | 5.4 | 53 | 2 | 3.8 | 46 | 1 | 2.2 |
| 11 | 47 | 2 | 4.3 | 82 | 3 | 3.7 | 56 | 0 | 0.0 |
| Total | 216 | 13 | 6.0 | 195 | 7 | 3.6 | 227 | 8 | 3.5 |

As misfit (or fit) cannot the assesses at the extremes, these are not included in the Table. Misfit of persons to the model is found for 6.0%, 3.6% and 3.5% in the respective scales. This is what is expected, and we therefore claim that the evidence shows that the Rasch model in each case fits all persons, and not just a large majority.
