# Supplementary material for: Psychometric evaluation of the Danish language version of the field practice experiences questionnaire for students in teacher education (FPE-DK) using item analysis according to the Rasch model
Source: PLoS One. 2021 Oct 18;16(10):e0258459. doi: 10.1371/journal.pone.0258459 (PMC8523040; doi:10.1371/journal.pone.0258459)
Supplement: S6 Table — (DOCX) [file pone.0258459.s008.docx]

**S6 Table. Weighted maximum likelihood estimates of person parameters for the three field work experience scales.**

|  | Observed scale | | | Practised scale | | | Received feedback scale | | |
| --- | --- | --- | --- | --- | --- | --- | --- | --- | --- |
| score^a^ | score SEM | PP | PP SEM | score SEM | PP | PP SEM | score SEM | PP | PP SEM |
| 0 | 0.64 | -3.568 | 0.862 | 0.65 | -3.500 | 0.843 | 0.65 | -3.467 | 0.844 |
| 1 | 1.05 | -2.300 | 0.844 | 1.07 | -2.276 | 0.823 | 1.07 | -2.240 | 0.822 |
| 2 | 1.28 | -1.623 | 0.783 | 1.30 | -1.627 | 0.771 | 1.31 | -1.593 | 0.768 |
| 3 | 1.44 | -1.121 | 0.708 | 1.45 | -1.143 | 0.702 | 1.47 | -1.114 | 0.697 |
| 4 | 1.55 | -0.702 | 0.655 | 1.55 | -0.731 | 0.653 | 1.57 | -0.710 | 0.647 |
| 5 | 1.61 | -0.326 | 0.625 | 1.61 | -0.356 | 0.626 | 1.62 | -0.344 | 0.619 |
| 6 | 1.63 | 0.029 | 0.614 | 1.62 | 0.004 | 0.617 | 1.64 | 0.006 | 0.610 |
| 7 | 1.62 | 0.379 | 0.620 | 1.61 | 0.363 | 0.625 | 1.63 | 0.355 | 0.618 |
| 8 | 1.57 | 0.742 | 0.645 | 1.55 | 0.736 | 0.651 | 1.57 | 0.718 | 0.645 |
| 9 | 1.48 | 1.140 | 0.693 | 1.46 | 1.145 | 0.700 | 1.47 | 1.118 | 0.694 |
| 10 | 1.32 | 1.608 | 0.760 | 1.31 | 1.624 | 0.768 | 1.32 | 1.591 | 0.763 |
| 11 | 1.08 | 2.241 | 0.812 | 1.07 | 2.268 | 0.821 | 1.08 | 2.227 | 0.817 |
| 12 | 0.66 | 3.450 | 0.836 | 0.65 | 3.491 | 0.841 | 0.65 | 3.447 | 0.840 |

PP = Person Parameter estimates. SEM = standard error of measurement

^a.^ Score range is the same for all three scales (0-12), so only shown once.
