# Supplementary material for: Psychometric evaluation of the Danish language version of the field practice experiences questionnaire for students in teacher education (FPE-DK) using item analysis according to the Rasch model
Source: PLoS One. 2021 Oct 18;16(10):e0258459. doi: 10.1371/journal.pone.0258459 (PMC8523040; doi:10.1371/journal.pone.0258459)
Supplement: S1 File — (DOCX) [file pone.0258459.s009.docx]

**S1. File. Description of competence objectives for field practice in teacher education**^[[1]](#footnote-1)^

Are concerned with 1) The practical and pedagogical dimension which is directed towards the teacher’s work with the pupils in school 2) The analytical dimension, which is concerned with the investigation of one’s own and other teachers’ practice. The subject of practice connects all the subjects at college with the students’ work at the practice schools.

Competence areas:

Competence area 1: Didactics

Competence area 2: Classroom management

Competence area 3: Relational work

**Competence area 1:** Didactics is about planning, implementing, evaluating and developing teaching

**Competence objectives:** By giving professional reasons and together with colleagues, the student can plan, implement, evaluate and develop teaching:

| Skills Objectives: the student can | Knowledge objectives: the student has knowledge about |
| --- | --- |
| *field practice level 1* |  |
| plan, set targets, implement and evaluate teaching sequences in cooperation with fellow students, | the aims and objectives of the Danish public school, principles for planning, teaching methods and the organization of pupils’ activities, taking their skills and learning abilities into consideration, |
| account for the effect of the teaching on the pupils’ learning, | types of evaluation and assessment of pupils’ learning at the practice school, |
| analyze teaching sequences with the purpose of developing teaching, | methods of observation, collection of data and documentation, |
| *field practice level 2* |  |
| plan, implement and evaluate a differentiated teaching course in cooperation with fellow students, making use of a variety of methods, including application-oriented forms of teaching and the inclusion of physical exercise in the lessons, | teaching methods, principles for differentiation, teaching resources and IT, |
| evaluate the teaching course and the pupils’ learning outcome, | formative and summative evaluation methods and tests, |
| observe own practice and the individual pupil’s learning in order to develop teaching, | methods of observation, collection of data and documentation, |
| *field practice level 3* |  |
| Plan, implement and evaluate a differentiated and long-term lesson plans in cooperation with fellow students and other resources while taking into consideration the overall curriculum for the year, | organisation methods, teaching methods and collaboration methods, |
| evaluate pupils’ learning outcomes and the overall effect of teaching during the unit. | formative and summative evaluation methods, |
| Develop and improve your own and others’ professional practice | methods of observation, collection of data and documentation. |

**Competence area 2:** Classroom Management is about the organization and development of the pupils’ academic and social learning environment

**Competence objectives:** The student can manage the classroom and establish and develop a clear and positive framework for the pupils’ learning and the social community of the class.

| Skills Objectives: the student can | Knowledge objectives: the student has knowledge about |
| --- | --- |
| *field practice level 1* |  |
| lead and manage pupils’ participation in the classroom teaching, | classroom management, |
| *field practice level 2* |  |
| develop a clear framework for learning and for the social life in the class in cooperation with the pupils and | classroom management, learning environment and the social relations in the class and |
| *field practice level 3* |  |
| lead inclusion processes in collaboration with students. | learning environment, inclusion, conflict management and bullying. |

**Competence area 3:** Relational work is about contact with and relations to pupils, colleagues, parents and the resource persons at the school

**Competence objectives:** The student can manage the positive cooperation with pupils, parents, colleagues and other resource persons and reflect upon the significance of relational work in concord with teaching and the pupils’ learning and well-being.

| Skills Objectives: the student can | Knowledge objectives: the student has knowledge about |
| --- | --- |
| *field practice level 1* |  |
| communicate with pupils to promote learning and well-being, | communication, pupils’ well-being, motivation, learning and relations with and amongst pupils, |
| communicate with parents about the teaching and the aims and objectives of school, | school-home cooperation, |
| *field practice level 2* |  |
| through a dialogic approach cooperate with students and colleagues about adjusting the teaching and the pupils’ active participation, | communication, engaging learning environments, motivation and well-being, |
| communicate in writing and orally with parents about aims and contents of planned teaching courses, | professional communication – oral and digital, |
| *field practice level 3* |  |
| support every pupil’s active participation in teaching and the social life of the class, collaboration with various parties at the school, and | communication that recognizes pupils, equal collaboration, and inclusion processes, |
| communicate with parents about their children’s participation and wellbeing at school. | processes that promote constructive collaboration between school and the pupils’ homes and forms of cooperation at parent meetings and contact groups |

1. The description of the competence areas, skills and knowledge objectives for the student field practice/internship are translation of the official text provided by the Danish Ministry of Education at: <https://www.retsinformation.dk/eli/lta/2015/1068#Bil3>. The translation to English was provided by the head of field practice at University College South from their international teacher education track. [↑](#footnote-ref-1)
